# EOAI3402143 inhibits lung adenocarcinoma progression through the NF-κB/NR4A1 pathway: EOAI3402143 inhibits lung adenocarcinoma through NF-κB/NR4A1

**DOI:** 10.3724/abbs.2025138

**Published:** 2025-10-09

**Authors:** Jia Xu, Wenjun Liu, Li Chen, Juan Zhang, Dongze Zhang, Haitao Huang, Xiaoming Zhang, Xue Huang, Guangbo Zhang

**Affiliations:** 1 Jiangsu Institute of Clinical Immunology the First Affiliated Hospital of Soochow University Suzhou 215000 China; 2 Jiangsu Key Laboratory of Clinical Immunology Soochow University Suzhou 215000 China; 3 School of Life Sciences Soochow University Suzhou 215123 China; 4 Shanghai Institute of Immunity and Infection Chinese Academy of Sciences Shanghai 200031 China; 5 Department of Thoracic Surgery the First Affiliated Hospital of Soochow University Suzhou 215006 China

**Keywords:** EOAI3402143, NR4A1, lung adenocarcinoma, NF-κB

## Abstract

Lung adenocarcinoma (LUAD) is currently the cancer with the highest morbidity and mortality rates in the world, and its targeted therapy, although effective, is limited in the types of targeted drugs and prone to drug resistance in treated patients. Therefore, the continuous discovery of new targeted therapeutic agents is particularly crucial for the treatment of LUAD. Here, we aim to investigate the antitumor effect of EOAI3402143 on LUAD and the potential mechanism of its action. We use flow cytometry to analyze apoptosis, transwell and colony formation assays to detect cell migration, invasion and proliferation ability; western blot, RT-qPCR and RNA-seq to analyze the signaling pathways involved in EOAI3402143; and *in vivo* experiments to test the therapeutic effect of EOAI3402143 on LUAD. Our results show that EOAI3402143 promotes apoptosis and inhibits the migration, invasion and proliferation of LUAD cells. Mechanistic studies reveal that EOAI3402143 inhibits the activation of the NF-κB pathway and suppresses the expression of NR4A1, which in turn inhibits the progression of LUAD. *In vivo* experiments reveal that EOAI3402143 has a better therapeutic effect on LUAD. These findings indicate that EOAI3402143 has significant antitumor efficacy against LUAD and is promising as a new therapeutic agent for LUAD.

## Introduction

Lung cancer is one of the common malignant tumors that threatens human life and health, with high morbidity and mortality. Lung cancer is mainly divided into non-small cell lung cancer (NSCLC) and small-cell lung cancer (SCLC), of which NSCLC accounts for approximately 80%–85% of cases [ 1– 3]. NSCLC includes lung squamous cell carcinoma (LUSC), lung adenocarcinoma (LUAD), and large cell carcinoma (LCC), of which lung adenocarcinoma is the cancer with the highest mortality rate among lung cancers [4]. Despite great advances in the diagnostic methods and treatment of LUAD in recent years, most patients experience local spread and distant metastases, and the prognosis remains poor, with a five-year survival rate of only approximately 15% [5]. This grim prognosis is due to advanced disease manifestations, heterogeneous tumor characteristics with multiple histological subtypes and our poor understanding of tumor biology [6]. A molecular classification of lung adenocarcinoma subgroups has been established, the most common of which are KRAS mutations and EGFR mutations [7]. Lung adenocarcinomas with different mutation types have different sensitivities to drugs; therefore, personalized therapeutic agents are needed for the treatment of lung adenocarcinomas. Although great progress has been made in targeted therapy for LUAD in recent years, some types of mutated lung adenocarcinomas for which there are no suitable targeted drugs exist, and even if there are, it is extremely common for patients to develop resistance to targeted drugs, and their five-year survival rate is still low [8]. After exhausting the available targeted agents, patients are usually treated with nontargeted, systemic cytotoxic regimens with unsatisfactory results [9]. Therefore, finding new potential therapeutic agents with antitumor efficacy and low toxicity for LUAD patients is particularly important to address this dilemma.

EOAI3402143 is a novel small-molecule compound that we identified through screening a 600-compound small-molecule library. As a typical small-molecule agent, EOAI3402143 can penetrate cell membranes and interact with specific intracellular targets. Small-molecule targeted drugs offer precise action against diseased cells while minimizing damage to normal cells, featuring high efficacy, low toxicity, and convenient administration, making them an important approach for personalized LUAD treatment. Current research on EOAI3402143 remains limited. Although existing studies have demonstrated its tumor-suppressive activity in NSCLC, the precise molecular mechanisms remain unclear. Our study aimed to elucidate these underlying mechanisms [ 10 – 12]. NR4A1 is a member of the NR4A superfamily, which is an important member of the nuclear receptor superfamily [13]. NR4A1 plays important roles in metabolism, the immune system, and cardiovascular and neurological functions ( *e.g.*, learning and memory and wound healing) [ 14– 20]. It has been shown that some agents induce NR4A1 nuclear export, which ultimately induces cell death [ 21– 25]. Interestingly, our RNA-seq analysis identified NR4A1 as one of the differentially expressed genes. The experimental results further demonstrated that EOAI3402143 significantly suppressed NR4A1 expression. On the basis of these findings, we hypothesize that EOAI3402143 may exert its antitumor activity through the downregulation of NR4A1. These findings were subsequently experimentally validated.

The aim of this study was to determine whether EOAI3402143 is effective against LUAD and to investigate the mechanism of action by which it exerts antitumor efficacy. Our study demonstrated that EOAI3402143 exerts antitumor effects on LUAD through the NF-κB/NR4A1 pathway, suggesting that EOAI3402143 could be a potential drug for the treatment of LUAD.

## Materials and Methods

### Cell lines

A549, H1299 and BEAS-2B cell lines were purchased from the Cell Bank of the Chinese Academy of Sciences (Shanghai, China), and all three cell lines were cultured in RPMI-1640 medium (C0224; Beyotime, Shanghai, China) supplemented with 10% fetal bovine serum (U16001DC; EallBio, Beijing, China) and 1% penicillin-streptomycin-amphotericin. The cells were placed in a humidified incubator at 37°C containing 5% CO _2_.

### Cell viability assay

Cell viability was evaluated using a CCK-8 kit (C6005; NCM Biotech, Suzhou, China) following the manufacturer’s protocol. Briefly, LUAD cells were seeded into 96-well plates at a density of 4000 cells per well and treated with varying concentrations of EOAI3402143 for 48 h. Subsequently, CCK-8 reagent was added to each well, and the plates were incubated at 37°C for 2 h. The absorbance was then measured at 450 nm, and the cell inhibition rate was calculated based on the obtained values.

### RT-qPCR

Total RNA was extracted from the cells via an RNA extraction kit (RC112-01; Vazyme, Nanjing, China), followed by cDNA synthesis of total RNA via a HiScript III RT SuperMix kit (R323-01; Vazyme). AceQ qPCR was performed via a SYBR Green premix (without ROX) kit (Q121-02-AA; Vazyme) for real-time quantitative PCR. Cycling conditions were: 95°C, 5 min; 95°C, 10 s; 60°C, 30s; 36 amplification cycles. The relative expression of genes was normalized to that of *GAPDH*, and the 2 ^–ΔΔCt^ method was used for the calculation of the relative expression of mRNAs. The sequences of primers used were as follows: *GAPDH* forward: 5′-CTCCTGCACCACCAACTGCT-3′, reverse: 5′-GGGCCATCCACAGTCTTCTG-3′; *NR4A1* forward: 5′-GGCTCGGGGATACTGGATACA-3′, reverse: 5′-CTGGCATGAAGCGTTGTCC-3′.

### Western blot analysis

Cells were lysed via RIPA lysis buffer (P0013B; Beyotime), and the lysate was sonicated on ice at 20% amplitude for 1 min and then centrifuged at 4°C for 20 min at 13,000 *g*, after which the supernatant was collected. The total protein concentration was measured via an Enhanced BCA Protein Assay Kit (P0009; Beyotime). Proteins were electrophoresed on 10% SDS-PAGE gel (P2012; NCM Biotech) and transferred onto PVDF membranes (10600023; GE, Uppsala, Sweden). The membranes were blocked with 5% skim milk (P0216; Beyotime) for 2.5 h at room temperature, after which the membranes were incubated with the indicated primary antibodies overnight at 4°C on a shaker. The membranes were then incubated with HRP-conjugated secondary antibodies (SA00001-1, SA00001-2, 1:5000; Proteintech, Wuhan, China) for 1 h. After the membranes were washed, the protein bands were detected with ECL luminescent solution (10100; NCM Biotech) and visualized via a Chemi Doc MP imaging system (Bio-Rad, Hercules, USA). The primary antibodies used were as follows: GAPDH mouse monoclonal antibody (10494-1-AP, 1:10,000; Proteintech), PARP (13371-1-AP, 1:1000; Proteintech), CL-PARP(60555-1-Ig, 1:5000; Proteintech) NR4A1 (12235-1-AP, 1:2000; Proteintech), CL-caspase9 (10380-1-AP, 1:1000; Proteintech), caspase9 (66169-1-Ig, 1:1000; Proteintech), p65 (10745-1-AP, 1:1000; Proteintech), and p-p65 (3033T, 1:2000; CST, Boston, USA).

### Cell transferection

Lentivirus was packaged in HEK293T cells by co-transfection of the transfer plasmid (pLV3-NR4A1-3×FLAG) (Miaoling, Wuhan, China) with the packaging plasmids (PSPA×2 and PMD2.G) (Miaoling). The transfection was performed using Polybrene (C0351; Beyotime) as the transfection reagent. The viral supernatant was collected, filtered, and subsequently used to infect both A549 and H1299 cell lines. Following infection, the transduced A549 and H1299 cells were subjected to selection with puromycin (P8230; Solarbio, Beijing, China) to establish stable polyclonal cell lines overexpressing NR4A1-3×FLAG. The overexpression of NR4A1 was rigorously validated at both the mRNA and protein levels. RT-qPCR analysis confirmed the elevated levels of NR4A1 mRNA transcripts. Furthermore, western blot analysis using an anti-FLAG antibody successfully detected the exogenous NR4A1-3×FLAG fusion protein, confirming successful protein expression.

### Flow cytometry

Cells were collected via EDTA-free trypsin digestion and resuspended in flow tubes with 100 μL of 1× apoptosis buffer. Then, 2.5 μL of 7-AAD/Annexin V-PE (559763; BD Biosciences, Franklin Lakes, USA) solution was added to each tube, the mixture was incubated at room temperature in the dark for 20 min, 300 μL of apoptosis buffer was added, and the rate of apoptosis was analyzed with a flow cytometer (Beckman Coulter, Brea, USA).

### RNA-seq analysis

A549 cells were treated with DMSO or EOAI3402143, and after 48 h, the cells were collected by trypsin digestion, resuspended in protective solution and sent to Bohao (Shanghai, China) for differential gene analysis, with three biological replicates per group. The data were then normalized, and genes with *P* < 0.05 were subjected to KEGG pathway analysis.

### Colony formation analysis

Cells were diluted and 1 mL of the cell suspension was added to a 12-well plate at 800 cells per well, which was subsequently changed to 1640 complete medium containing 1% FBS. After 7 days, the mixture was treated with the corresponding concentration of EOAI3402143. After 14 days, the medium was discarded, the cells were washed once with PBS, fixed with 4% paraformaldehyde for 20 min, washed once with PBS, stained with crystal violet for 20 min, washed once with PBS, and imaged to count the number of colonies.

### Transwell assay

For the migration assay, 800 μL of 1640 medium containing 10% FBS was added to the bottom chamber of 24-well plates, and 200 μL of cell suspension with 2.5 × 10 ^4^ cells in serum free medium was added to the upper chambers of 8 μm pore size inserts (353097; Corning, New York, USA). After 24 h, the A549 cells were fixed and stained with crystal violet, and the H1299 cells were fixed and stained after 40 h and photographed with an inverted microscope. For the invasion assay, 100 μL of stromal gel (353097; Corning) diluted 1:8 with 1640 medium was added to a small chamber and then placed in the incubator to solidify for 1 h, after which the medium was aspirated. Then, the cell suspension was added, and the remaining steps were the same as those of the migration assay.

### Tumor xenografts

BALB/c nude male nude mice (6-week-old) were purchased from Shanghai Lingchang Co., Ltd (Shanghai, China). Each mouse was injected with 5 × 10 ^6^ H1299 cells subcutaneously near the axilla. After 10 days, the mice were observed to have tumors 5 mm in diameter, and the mice were randomly divided into three groups with four mice in each group. The mice were treated with 5 mg/kg or 10 mg/kg EOAI3402143, and the control group was treated with DMSO, which was injected every two days. The body weights and tumor volumes of the mice were measured every two days, and the values were calculated as 1/2(L × W ^2^). After ten treatments, blood was taken from the orbital region of each mouse, and the mice were subsequently sacrificed by euthanasia. The heart, liver, spleen, lungs, and kidneys of the mice were collected and fixed in 4% paraformaldehyde. Subsequently, hematoxylin and eosin (H&E) staining was performed, and images were captured under a microscope. The study was conducted according to the guidelines of the First Affiliated Hospital of Soochow University and approved by the Laboratory Animal Ethics Committee of Soochow University (approval No. 202409A0963).

### Serum biochemical analysis

Blood samples were collected via the orbital venous plexus from anesthetized mice using heparinized capillary tubes. After clotting at room temperature for 30 min, samples were centrifuged at 3000 *g* for 15 min to separate the serum. The activities of alkaline phosphatase (AKP), alanine aminotransferase (ALT), and aspartate aminotransferase (AST) were measured using commercial assay kits (K422/K752/K753; Biovision, Beijing, China) following the manufacturer’s protocols. All measurements were performed in triplicate using a microplate reader.

### Small-molecule library

We purchased a small molecule compound library from MCE, with the library ID HY-LD-000004971-1 (MCE, Monmouth Junction, USA). This library contains 600 small-molecule drugs. For detailed information, please refer to Supplementary Table S1.

### Statistical analysis

Data were statistically analyzed via GraphPad Prism (9.0). Each experiment in this study was conducted in triplicate. Student’s *t* test was used to compare two groups, and ANOVA was used to compare multiple groups. Comparisons were made with the corresponding controls, and statistical significance was indicated significant by *P* < 0.05.

## Results

### EOAI3402143 inhibits LUAD cell viability

**Figure 1:**
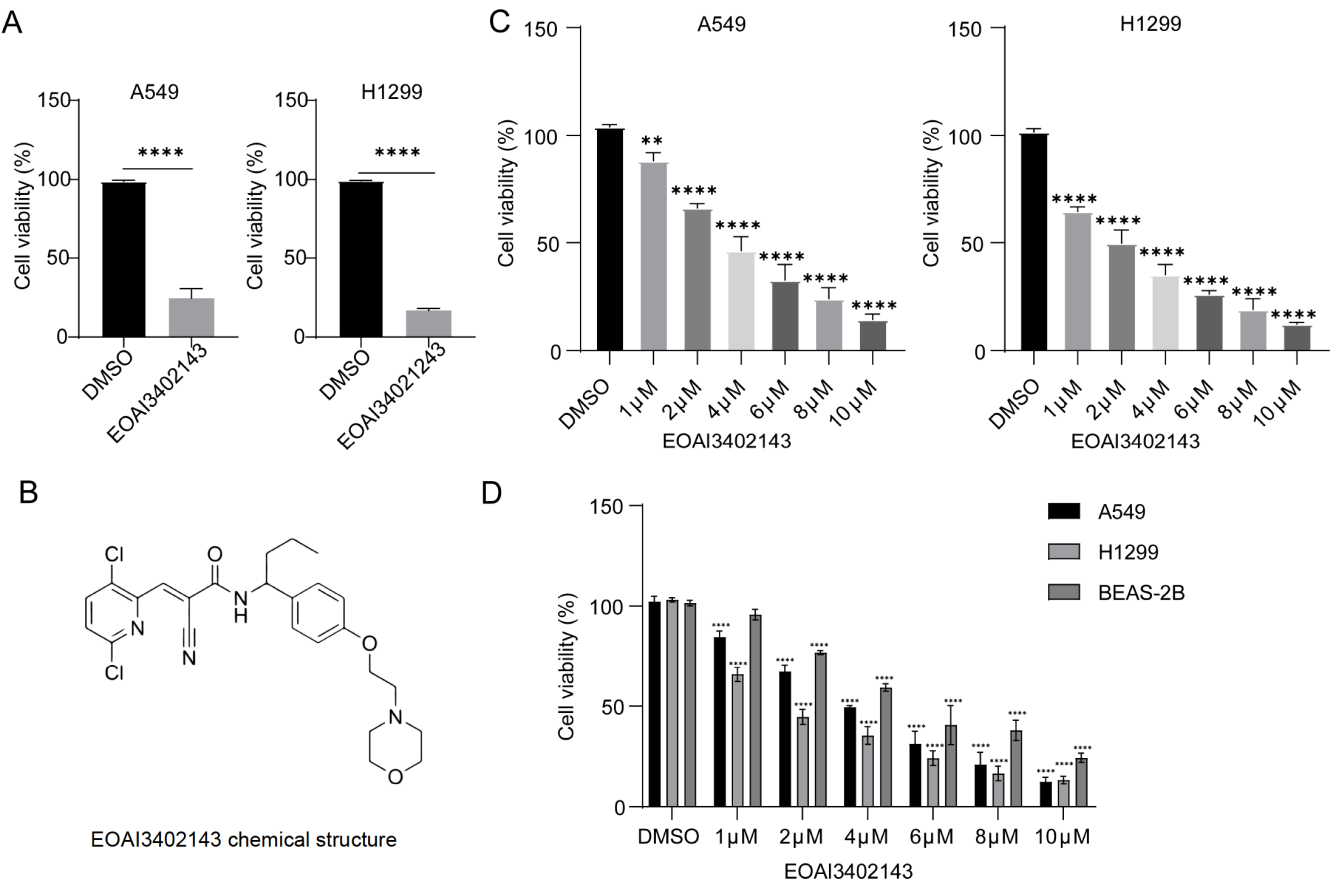
Figure 1 EOAI3402143 inhibits the viability of LUAD cells (A) Preliminary screening of EOAI3402143-mediated inhibition of both the A549 and H1299 cell lines. (B) Structural formula of EOAI3402143. (C) Viability of A549 and H1299 cells after treatment with different concentrations of EOAI3402143. (D) Viability of A549, H1299 and BEAS-2B cells after treatment with different concentrations of EOAI3402143. Each experiment was performed in triplicate. Data are presented as the mean ± SD. **P < 0.01, ****P < 0.0001.

To identify potential small-molecule inhibitors for lung adenocarcinoma treatment, we screened a commercially available library comprising 600 bioactive small-molecule compounds. Using a standardized concentration of 10 μM, we systematically evaluated the cytotoxic effects of each compound on A549 and H1299 cells cultured in 96-well plates. Cell viability was assessed by CCK-8 assay, with compounds demonstrating > 50% growth inhibition selected for further investigation in both cell lines. Through this screening approach, we identified EOAI34021243 as a promising candidate on the basis of its potent antitumor activity ( Figure 1A,B) and relatively unexplored mechanism of action in the current literature. CCK8 experiments revealed that EOAI3402143 had a greater inhibitory effect on A549 and H1299 cells in a concentration-dependent manner ( Figure 1C). Safety validation was subsequently performed in BEAS-2B cells ( Figure 1D).

### EOAI3402143 promotes apoptosis in LUAD cells

**Figure 2:**
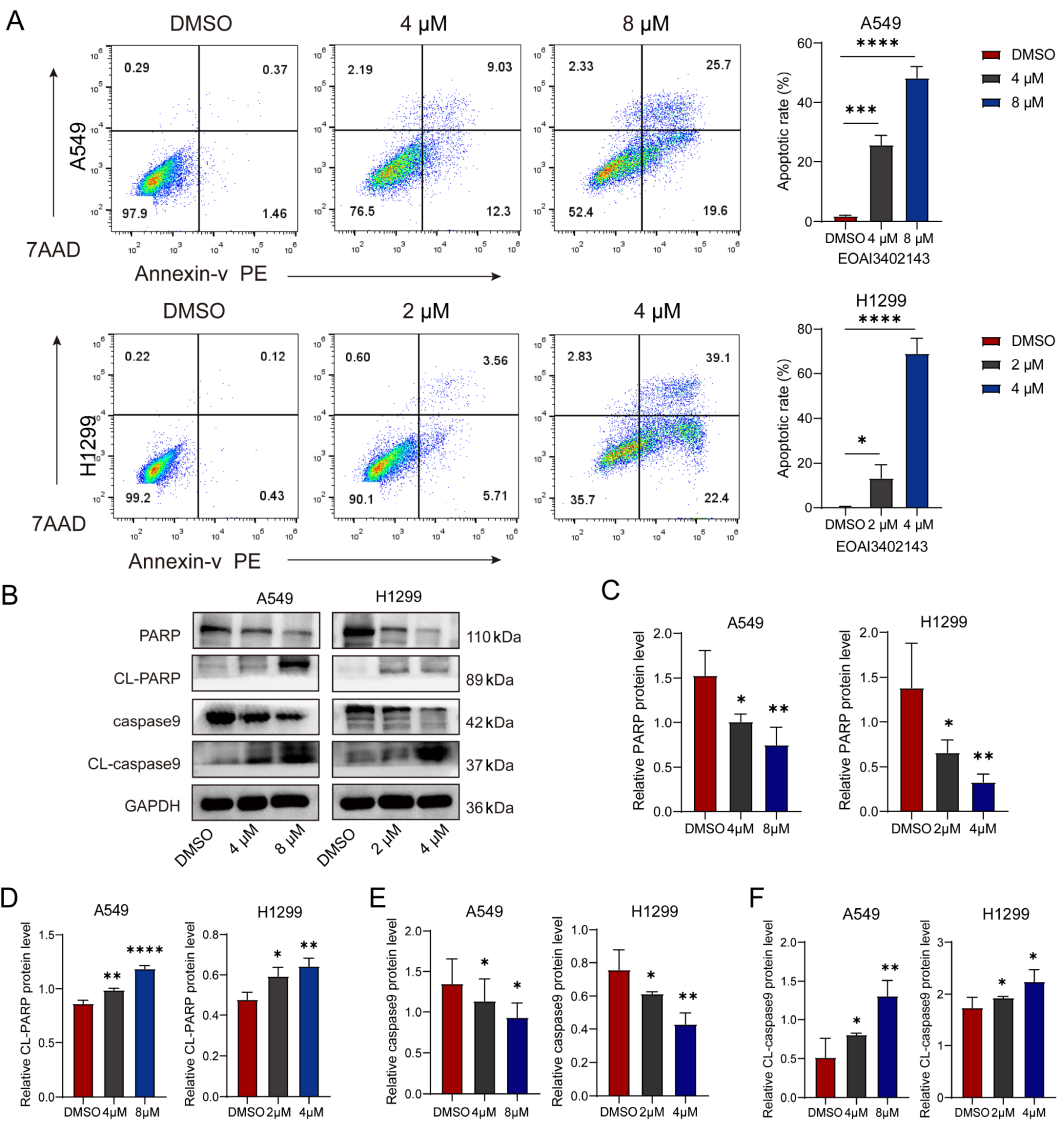
Figure 2 EOAI3402143 promotes apoptosis in LUAD cells (A) Flow cytometry analysis of the apoptosis rates of A549 and H1299 cells after treatment with DMSO or different concentrations of EOAI3402143. (B) Western blot analysis of the protein expression levels of PARP, CL-PARP, caspase9, and CL-caspase9 in A549 and H1299 cells after treatment with DMSO or different concentrations of EOAI3402143. (C) Densitometric analysis of PARP normalized to GAPDH. (D) Densitometric analysis of CL-PARP normalized to GAPDH. (E) Densitometric analysis of caspase9 normalized to GAPDH. (F) Densitometric analysis of caspase9 normalized to GAPDH. Each experiment was performed in triplicate. Data are presented as the mean ± SD. *P < 0.05, **P < 0.01, ***P < 0.001, ****P < 0.0001.

To further validate the antitumor effect of EOAI3402143 on A549 and H1299 cells, we detected the apoptosis rate of A549 and H1299 cells by flow cytometry after EOAI3402143 treatment. Compared with that of the DMSO group, the apoptosis rates of A549 and H1299 cells increased after EOAI3402143 treatment, especially at high concentrations ( Figure 2A). The western blot analysis results revealed that the expression levels of CL-PARP and CL-caspase9 apoptotic proteins were promoted after EOAI3402143 treatment ( Figure 2B–F). The above data indicated that EOAI3402143 promoted apoptosis in LUAD cells in a concentration-dependent manner.

### EOAI3402143 inhibits the migration, invasion and colony formation of LUAD cells

**Figure 3:**
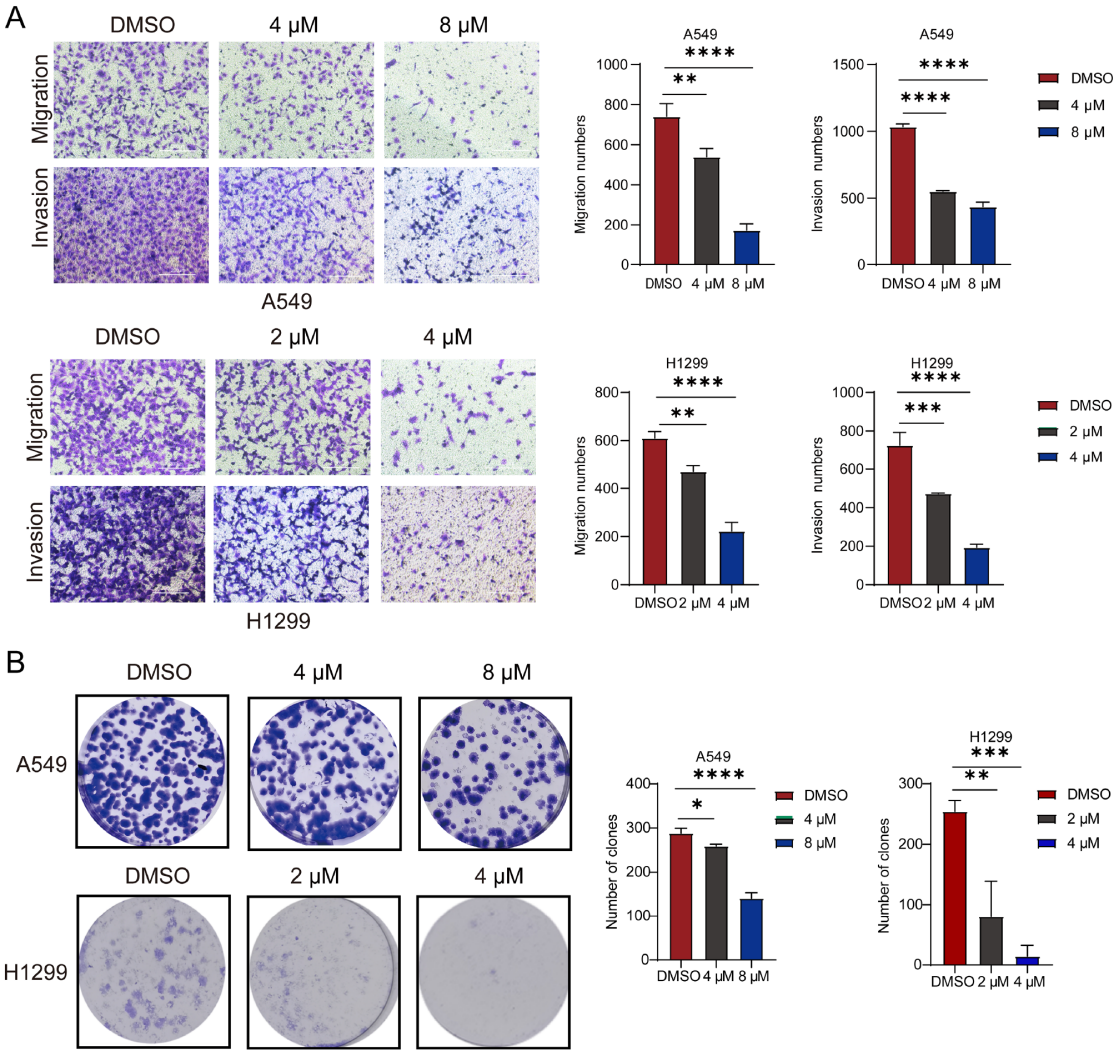
Figure 3 EOAI3402143 inhibits the migration, invasion and colony formation of LUAD cells (A) Transwell assays were used to analyze the migration and invasion ability of A549 and H1299 cells after DMSO treatment and treatment with different concentrations of EOAI3402143. (B) Colony formation assay to analyze the colony formation ability of A549 and H1299 cells after DMSO treatment and treatment with different concentrations of EOAI3402143. Each experiment was performed in triplicate. Data are presented as the mean ± SD. *P < 0.05, **P < 0.01, ***P < 0.001, ****P < 0.0001.

EOAI3402143 also inhibited the migration and invasion of LUAD cells. The control group was treated with DMSO, and the experimental group was treated with two concentrations of EOAI3402143. The results of the transwell assays revealed that the migration and invasion abilities of A549 and H1299 cells in the EOAI3402143 group were lower than those in the control group. A high concentration of EOAI3402143 better inhibited the migration and invasion of A549 and H1299 cells ( Figure 3A). Colony formation assay revealed that the colony formation ability of A549 and H1299 cells was significantly lower after EOAI3402143 treatment than after DMSO treatment ( Figure 3B). The above results indicated that EOAI3402143 inhibited the migration, invasion and colony formation of LUAD cells.

### EOAI3402143 inhibits the NF-κB pathway

**Figure 4:**
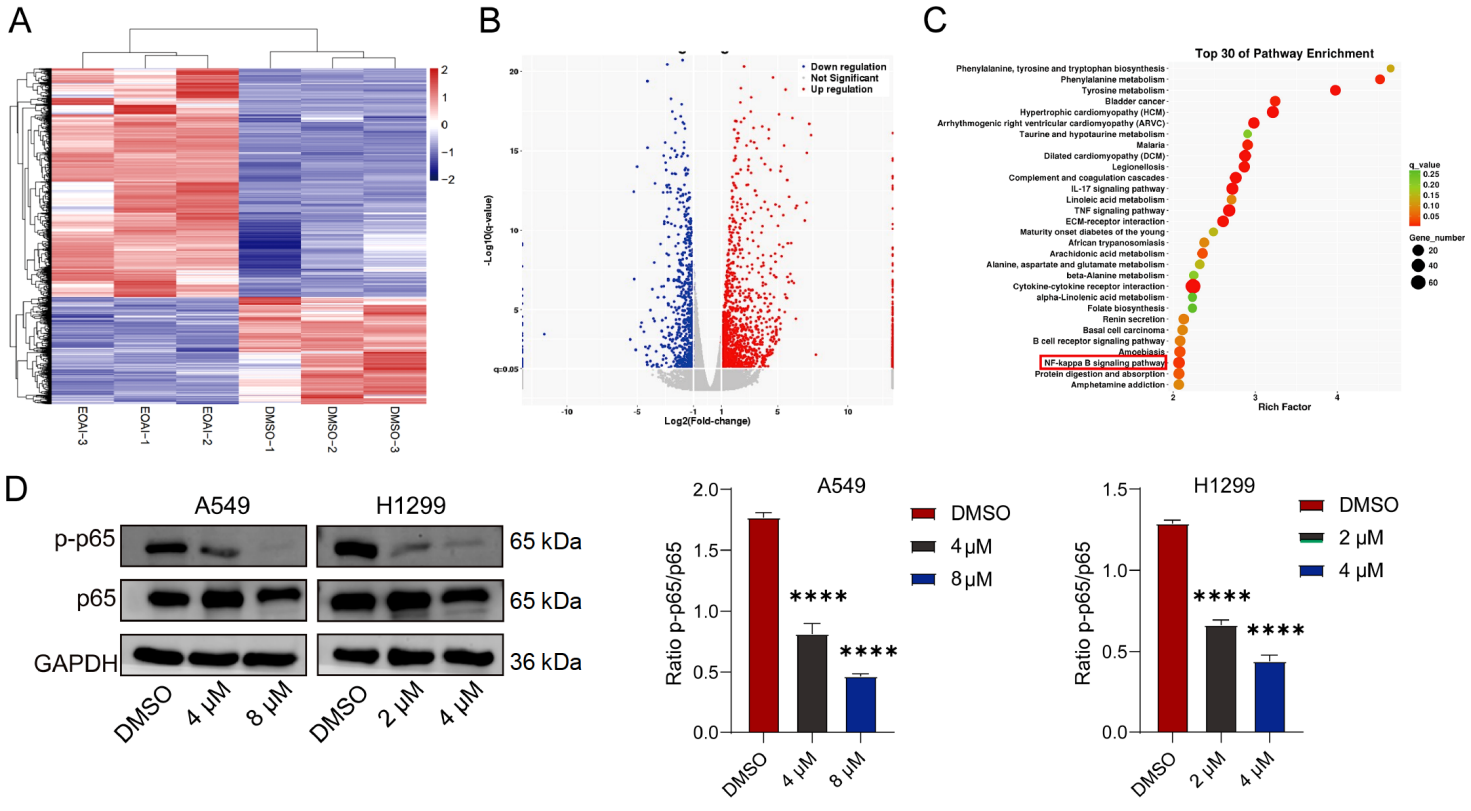
Figure 4 EOAI3402143 inhibits the NF-κB pathway (A) Heatmap showing differentially expressed genes at the transcriptome level in DMSO- and EOAI3402143-treated A549 cells according to the RNA-seq data. (B) Volcano plot showing differentially expressed genes at the mRNA level in the transcriptomes of DMSO- and EOAI3402143-treated A549 cells according to the RNA-seq data. (C) KEGG pathway enrichment analysis of the top 30 pathways enriched with DEGs after DMSO or EOAI3402143 treatment. (D) Western blot analysis of P65 and P-P65 protein levels after DMSO or EOAI3402143 treatment. Each experiment was performed in triplicate. Data are presented as the mean ± SD. ****P < 0.0001.

To investigate the mechanism by which EOAI3402143 affects the proliferation, apoptosis, migration and invasion of LUAD cells, A549 cells after treatment with DMSO or EOAI3402143 were subjected to transcriptomics analysis. The sequencing results revealed that 724 genes were downregulated and 1557 genes were upregulated after EOAI3402143 treatment compared with those in the DMSO group ( Figure 4A,B). We performed Kyoto Encyclopedia of Genes and Genomes (KEGG) enrichment of the pathways associated with these DEGs and found that many genes were associated with the NF-κB pathway ( Figure 4C). EOAI3402143 inhibited the activation of the NF-κB pathway ( Figure 4 D).

### EOAI3402143 promotes LUAD apoptosis by inhibiting NR4A1

**Figure 5:**
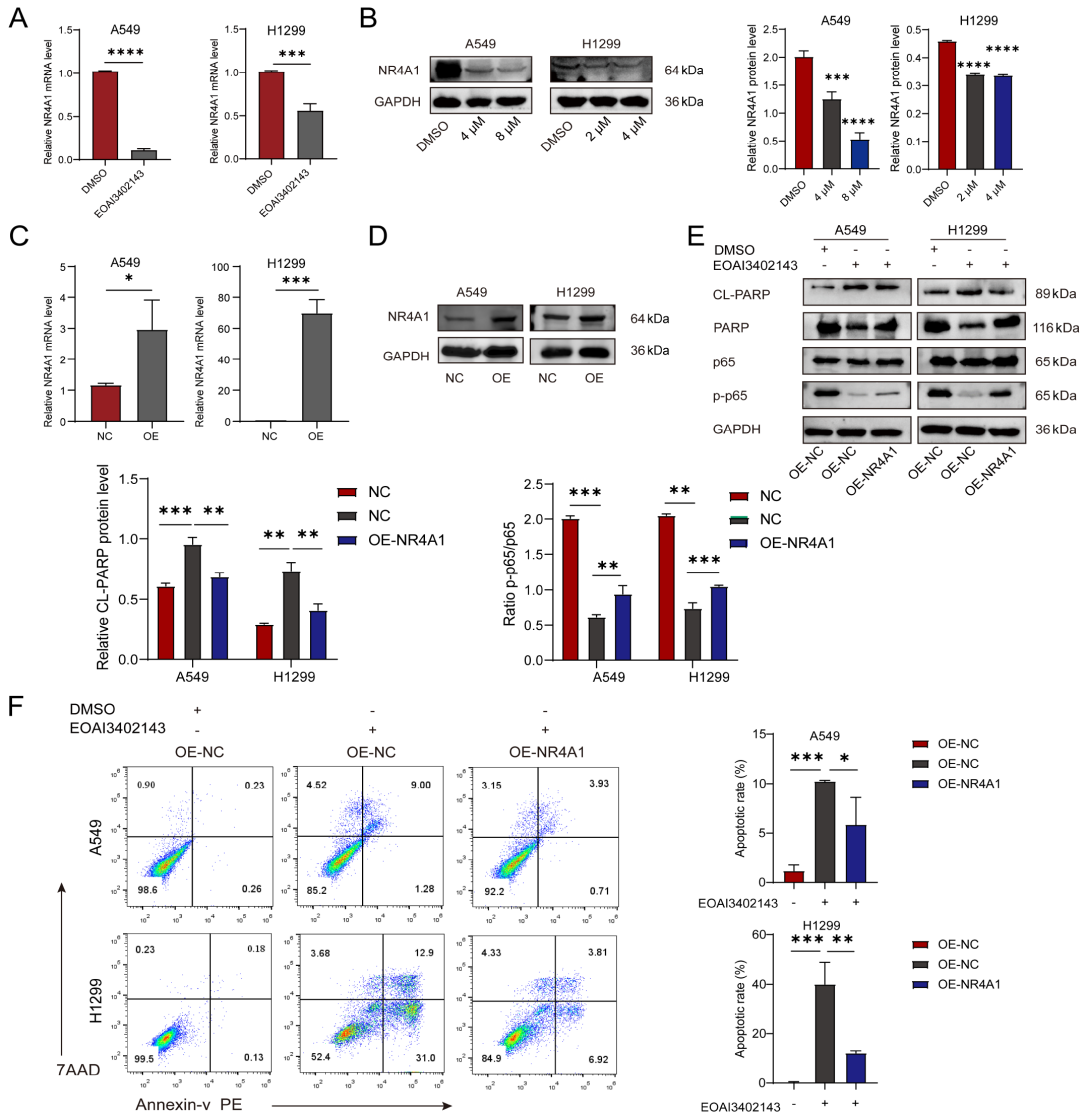
Figure 5 EOAI3402143 promotes LUAD apoptosis by inhibiting NR4A1 (A) RT-qPCR was used to analyze the mRNA expression level of NR4A1 in A549 and H1299 cells after DMSO or EOAI3402143 treatment. (B) Western blot analysis of the protein expression level of NR4A1 in A549 and H1299 cells after DMSO or EOAI3402143 treatment. (C) RT-qPCR was used to analyze the mRNA expression level of NR4A1 in A549 and H1299 cells after OE-NC and OE-NR4A1. (D) Western blot analysis of the protein expression levels of NR4A1 in A549 and H1299 cells. (E) Western blot analysis of the protein expression levels of CL-PARP and P-P65in A549 and H1299 cells treated with DMSO or EOAI3402143 after OE-NR4A1. (F) Flow cytometry analysis of the apoptosis rates of A549 and H1299 cells treated with DMSO or EOAI3402143 after OE-NR4A1. Each experiment was performed in triplicate. Data are presented as the mean ± SD. *P < 0.05, **P < 0.01, ***P < 0.001.

To further investigate the mechanism by which EOAI3402143 inhibits tumor development, we searched for differentially expressed genes through UniProt and PubMed, and finally obtained 8 candidate genes after understanding the biological processes in which these genes are involved as well as their gene properties. We verified these genes via RT-qPCR, and only *NR4A1* met expectations. The RNA-seq results revealed that NR4A1 is involved in the NF-κB pathway. After treatment with EOAI3402143, the mRNA level of *NR4A1* decreased in A549 and H1299 cells, which was consistent with the sequencing results ( Figure 5A). Western blot analysis revealed that the protein level of NR4A1 also decreased after EOAI3402143 treatment ( Figure 5B). To determine whether NR4A1 is involved in the inhibitory effect of EOAI3402143 on LUAD cells, we overexpressed NR4A1 in A549 and H1299 cells ( Figure 5C,D), and after treatment with EOAI3402143, western blot analysis revealed that the overexpression of NR4A1 inhibited the promotion of CL-PARP apoptotic protein expression induced by EOAI3402143 ( Figure 5E). The flow cytometry results revealed that NR4A1 overexpression inhibited the proapoptotic effect of EOAI3402143 on A549 and H1299 cells ( Figure 5 F).

### EOAI3402143 inhibits the migration, invasion and colony formation of LUAD cells by inhibiting NR4A1

**Figure 6:**
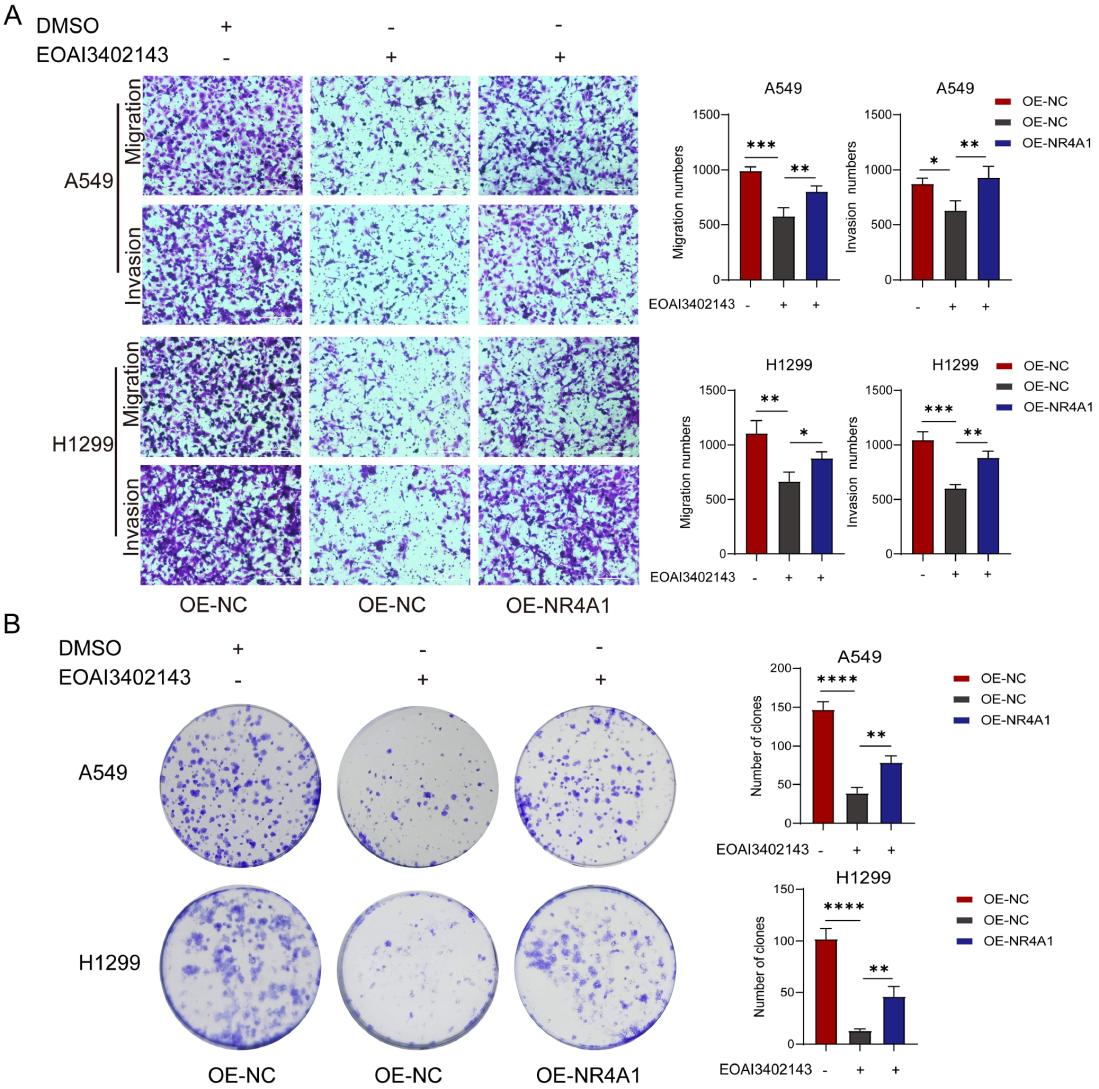
Figure 6 EOAI3402143 inhibits the migration, invasion and colony formation of LUAD cells by inhibiting NR4A1 (A) Transwell assays were used to analyze the migration and invasion ability of A549 and H1299 cells after OE-NR4A1 was added, followed by treatment with DMSO or EOAI3402143. (B) Colony formation assay was used to analyze the colony formation ability of A549 and H1299 cells after OE-NR4A1 was added, followed by treatment with DMSO or EOAI3402143. Each experiment was performed in triplicate. Data are presented as the mean ± SD. *P < 0.05, **P < 0.01, ***P < 0.001, ****P < 0.0001.

Migration and invasion assays revealed that the inhibitory effect of EOAI3402143 on the migratory ability of LUAD cells could be reversed by NR4A1 ( Figure 6A). Colony formation assay revealed that NR4A1 overexpression reversed the inhibitory effect of EOAI3402143 on the colony formation of A549 and H1299 cells ( Figure 6B). These results indicated that NR4A1 is able to attenuate the antitumor effect of EOAI3402143 on LUAD cells. The above experiments demonstrated that EOAI34021243 inhibited LUAD cell colony formation, migration and invasion through the NF-κB/NR4A1 pathway.

### *In vivo* antitumor activity of EOAI3402143

**Figure 7:**
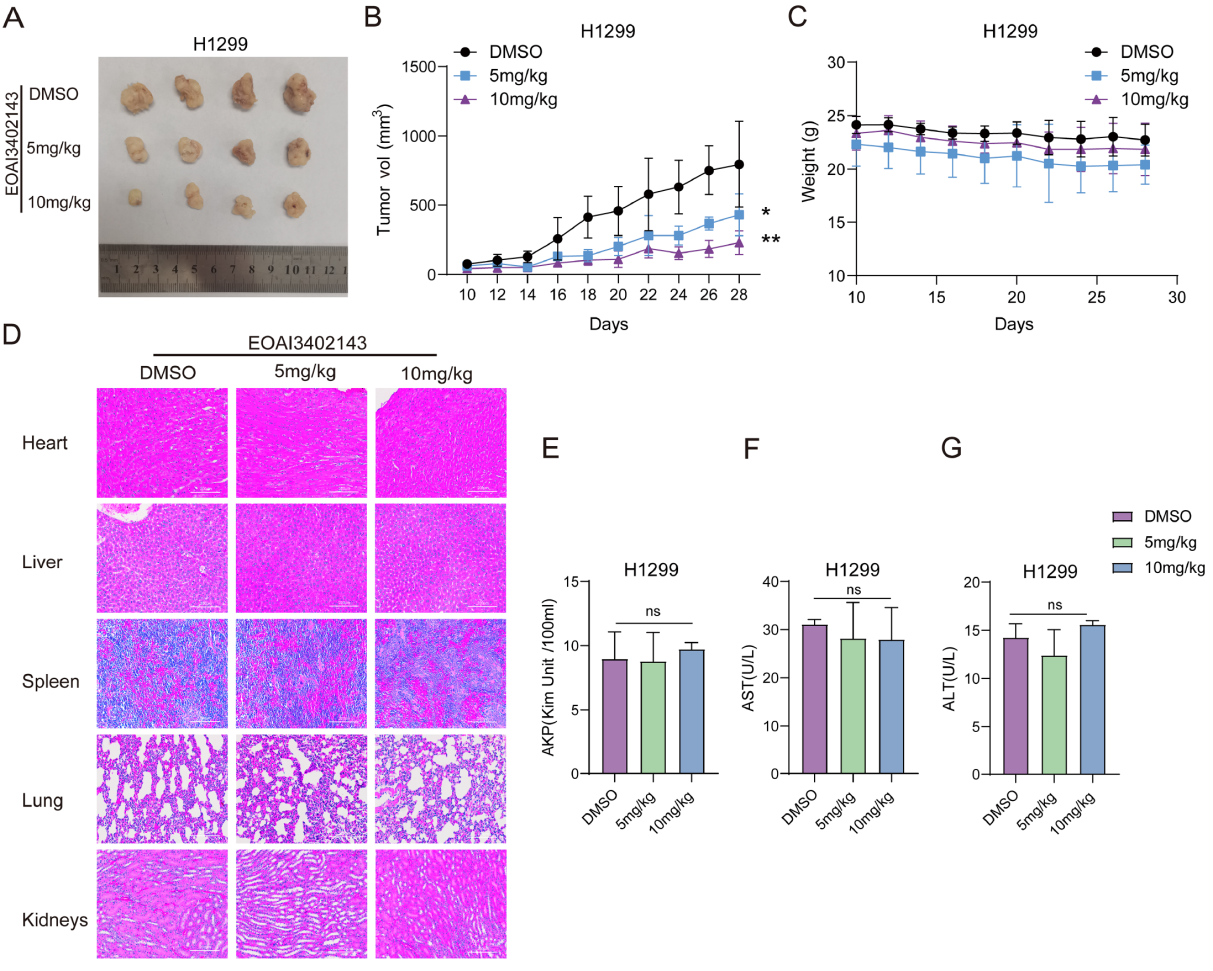
Figure 7 *In vivo* antitumor activity of EOAI3402143 (A) Tumor images after treatment with DMSO and two concentrations of EOAI3402143 following subcutaneous injection of H1299 cells. (B) Tumor growth after regular injection of DMSO or two concentrations of EOAI3402143. (C) Body weight changes in mice injected regularly with DMSO or both concentrations of EOAI3402143. (D) HE-stained images of the hearts, livers, spleens, lungs and kidneys of the mice in the DMSO group and in the groups treated with both concentrations of EOAI3402143. (E) Serum levels of AKP in the mice in the DMSO group and the two concentrations of EOAI3402143 group. (F) AST levels in the serum of the mice in the DMSO group and the two groups treated with different concentrations of EOAI3402143. (G) Levels of ALT in the serum of the mice in the DMSO group and the two concentrations of EOAI3402143 group. Each experiment was performed in triplicate. Data are presented as the mean ± SD. *P < 0.05, **P < 0.01, ****P < 0.0001. ns, not significant.

To verify the effect of EOAI3402143 *in vivo*, we performed experiments in 6-week-old BALB/c male nude mice. The results showed that the tumors of the nude mice treated with 10 mg/kg EOAI3402143 were smaller than those of the 5 mg/kg group, and all of them were smaller than those of the DMSO group ( Figure 7A,B). Thus, EOAI3402143 can inhibit the proliferation of LUAD cells in nude mice, which suggests that EOAI3402143 is a potential drug for the treatment of LUAD. After that, to verify the safety of this drug, we detected weight changes in the nude mice, and the results revealed that there was no significant difference in the body weights of the three groups of nude mice ( Figure 7C). Moreover, we performed HE staining of the heart, liver, spleen, lung and kidney of the three groups of nude mice, and the results revealed that there were no pathological changes ( Figure 7D). In addition, we also detected the serum levels of ALT, AST, and AKP in the three groups of nude mice, and the results revealed that there were no significant differences in these three indices after treatment with EOAI3402143 ( Figure 7E–G). These findings indicate that EOAI3402143 has no toxic effect on nude mice. In summary, EOAI3402143 could be a potential drug for the treatment of LUAD.

## Discussion

According to global cancer statistics from 2020, the incidence of lung cancer is the second highest, and lung cancer is the leading cause of cancer-related mortality [26]. Among lung cancers, lung adenocarcinoma accounts for a high proportion of cases, and early diagnosis is difficult for cases who are usually found to be in the advanced stage and more difficult to cure. At the same time, patients are prone to develop resistance to targeted drug therapy. Thus, the discovery of new antitumor and low-toxicity drugs is particularly important for the treatment of lung adenocarcinoma [1].

In recent years, an increasing number of small-molecule drugs have been found to have significant antitumor efficacy. Recently, we reported that EOAI3402143 had significant antitumor activity against LUAD during the screening of small molecule drugs; therefore, we speculated that EOAI3402143 might serve as a potential therapeutic agent for LUAD. It has been shown that EOAI3402143 can inhibit tumor growth *in vivo* [11]. Our experiments demonstrated that EOAI3402143 inhibited the proliferation, migration, and invasion of LUAD cells in a dose-dependent manner and promoted the apoptosis of LUAD cells. These results indicate that EOAI34021243 has antitumor efficacy against LUAD. Our *in vivo* experiments in mice also revealed that EOAI3402143 inhibited the growth of LUAD solid tumors and that there was no significant change in the body weight of the mice after treatment, which suggests that EOAI3402143 has low toxicity and possesses a certain degree of safety. These findings indicate that EOAI3402143 is a promising new drug for the treatment of LUAD patients.

To investigate the mechanisms by which EOAI3402143 exerts its antitumor activity, we performed transcriptomic analysis. KEGG pathway enrichment analysis of our sequencing results revealed that EOAI3402143 may inhibit tumor growth through the NF-κB pathway. The NF-κB pathway has long been recognized as a key mediator that promotes inflammatory responses. Tumorigenic pathogens can lead to infections with chronic inflammation, and long-term accumulation can lead to malignant tumors [ 27– 30]. The activation of the NF-κB pathway, which is the body’s response to resisting the invasion of pathogens, also promotes the growth, proliferation, migration and invasion of tumor cells as well as angiogenesis, which in turn facilitates the process of tumor development. Therefore, inhibitors that block NF-κB may be used in clinic for the treatment of tumors [31]. Several anti-inflammatory drugs, such as aspirin, sodium salicylate and dexamethasone, have been shown to inhibit NF-κB activation [ 32, 33]. Our experimental validation revealed a significant decrease in P-P65/P65 in A549 and H1299 cells after EOAI3402143 treatment, suggesting that EOAI3402143 inhibits NF-κB activation and may exert antitumor activity by inhibiting the NF-κB pathway.

After that, we analyzed the genes with large differential values in the sequencing results, and combined with a literature search as well as experimental screening, we finally identified the gene *NR4A1*. NR4A1 acts as a tumor promoter in most tumors. NR4A1 is a member of the nuclear receptor subfamily 4. *NR4A1* is a gene that is stimulated by stress, cytokines, growth factors, glucose, fatty acids, or other stimuli induced by immediate genes [ 34– 37]. NR4A1 plays multiple roles in many physiological and pathological processes, such as cell survival, apoptosis, differentiation, cell cycle, inflammation, immunity and metabolism [ 38– 42]. NR4A1 binds to Sp1 and p300 in the survivin gene promoter region, increasing pancreatic cancer cell proliferation and decreasing apoptosis [ 43, 44]. Resveratrol binds to nuclear receptor 4A1 (NR4A1) and acts as an NR4A1 antagonist in lung cancer cells [45]. NR4A1 is also induced by nuclear export and subsequently interacts with a variety of mitochondrial and extramitochondrial factors, leading to the induction of cell death [24]. Therefore, we speculated that EOAI3402143 likely exerts its effects by regulating NR4A1. This was indeed demonstrated in our study. Our transcriptome sequencing results revealed that the mRNA expression level of *NR4A1* decreased after EOAI3402143 treatment. After experimental validation, the mRNA expression level of *NR4A1* decreased after the addition of EOAI3402143, and the protein expression level also decreased. It is thus clear that EOAI3402143 may exert its antitumor activity by inhibiting NR4A1. Next, to verify that NR4A1 is actually involved in this process, we overexpressed NR4A1 and then treated it with the same concentration of EOAI3402143. The experimental results revealed that after NR4A1 was overexpressed, the inhibitory effects of EOAI3402143 on the proliferation, migration, and invasion of A549 and H1299 cells and the promotion of the apoptosis of A549 and H1299 cells were suppressed. EOAI34021243 exerts its antitumor effects through NR4A1. However, this study did not validate whether NR4A1 is the target of EOAI3402143, which is a challenge, and further research is needed in the future.

**Figure 8:**
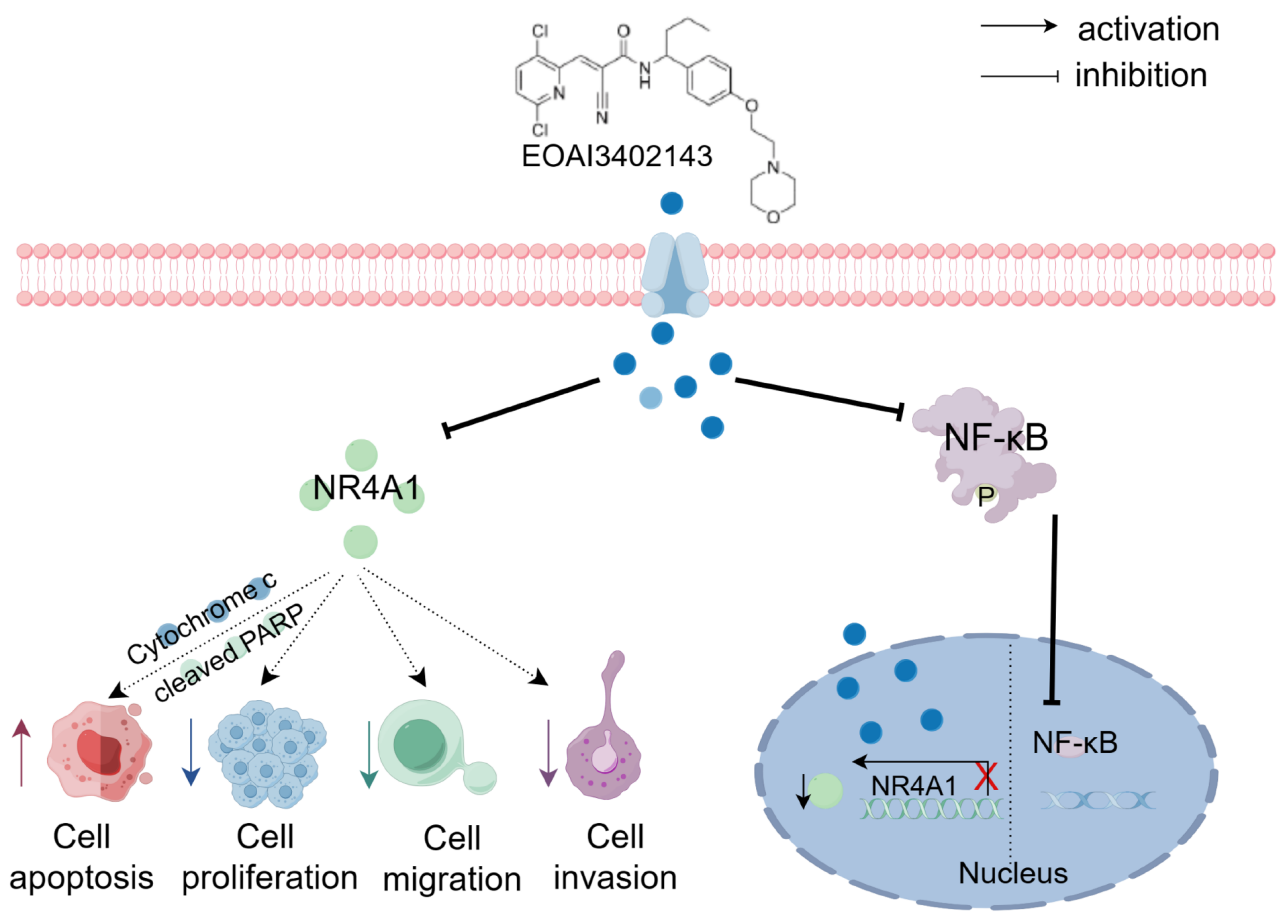
Figure 8 Schematic diagram of the proposed mechanism by which EOAI3402143 inhibits the progression of LUAD through the NF-κB/NR4A1 pathway

In summary, we determined that EOAI3402143 can be used as an antitumor drug. EOAI3402143 promotes apoptosis and inhibits the proliferation, migration, and invasion of LUAD cells through the NF-κB/NR4A1 pathway, which in turn inhibits the progression of LUAD ( Figure 8). These findings indicate that EOAI3402143 could be a potential drug for the treatment of LUAD.

## Supporting information

25283TabS1

## Supplementary Data

Supplementary data is available at *Acta Biochimica et Biophysica Sinica* online.
