# Supplementary material for: EOAI3402143 inhibits lung adenocarcinoma progression through the NF-κB/NR4A1 pathway: EOAI3402143 inhibits lung adenocarcinoma through NF-κB/NR4A1
Source: Acta Biochim Biophys Sin (Shanghai). 2025 Oct 9;58(4):833–44. doi: 10.3724/abbs.2025138 (PMC13107016; doi:10.3724/abbs.2025138)
Supplement: 25283TabS1 [file 25283TabS1.docx]

**Supplementary Table S1. Information of 600 small molecule drugs**

| Catalog No. | Product name | M.Wt | |
| --- | --- | --- | --- |
| HY-B1368 | Fenclonine | 199.63 | |
| HY-112831 | Osoresnontrine | 311.34 | |
| HY-100771 | PCC0208009 | 497.63 | |
| HY-19617B | EGFR-IN-1 (TFA) | 628.60 | |
| HY-10593 | IQ 1 | 362.42 | |
| HY-101867 | AU1235 | 324.34 | |
| HY-105917 | Emidurdar | 495.18 | |
| HY-13569A | Beraprost (sodium) | 420.48 | |
| HY-100943 | Cinanserin (hydrochloride) | 376.94 | |
| HY-117048 | PF-4191834 | 393.50 | |
| HY-100944 | Conduritol B epoxide | 162.14 | |
| HY-43533 | Tarlox-TKI | 461.74 | |
| HY-13427 | Allitinib tosylate | 621.08 | |
| HY-108676 | NF023 (hexasodium) | 1162.88 | |
| HY-100335 | PCI-33380 | 855.78 | |
| HY-111634 | Epsilon-momfluorothrin | 385.35 | |
| HY-101771A | (2-Chloropyridin-4-yl)methanamine hydrochloride | 179.05 | |
| HY-101266B | Milademetan (tosylate hydrate) | 808.74 |  |
| HY-101294 | TPPU | 359.34 |  |
| HY-N5024 | Gambogenic acid | 630.77 |  |
| HY-116009 | RMC-4550 | 437.36 |  |
| HY-111540B | (S)-LY-3381916 | 396.45 |  |
| HY-N0110 | Palmatine (chloride) | 387.86 |  |
| HY-112287 | ERK1/2 inhibitor 1 | 550.05 |  |
| HY-121186 | Bevantolol (hydrochloride) | 381.89 |  |
| HY-106840 | L-365260 | 398.46 |  |
| HY-107364 | MTX-211 | 478.33 |  |
| HY-17452A | Cefditoren (Pivoxil) | 620.72 |  |
| HY-116856 | SRPKIN-1 | 472.53 |  |
| HY-13623 | Entecavir | 277.28 |  |
| HY-105284 | Sulopenem | 349.45 |  |
| HY-112077 | (Z)-Viaminate | 447.61 |  |
| HY-101474B | (R)-Zanubrutinib | 471.55 |  |
| HY-106446 | Rentiapril | 313.39 |  |
| HY-10038 | A 922500 | 428.48 |  |
| HY-116814 | ML401 | 419.74 |  |
| HY-13036 | (Rac)-IBT6A | 386.45 |  |
| HY-116010 | Oleandomycin | 687.86 |  |
| HY-111101 | AZ1495 | 385.50 |  |
| HY-10836 | Cruzain-IN-1 | 300.27 |  |
| HY-107442 | PROTAC BRD4-binding moiety 1 | 371.43 |  |
| HY-134761 | EN4 | 416.47 |  |
| HY-119293 | K777 | 574.73 |  |
| HY-19314 | Azvudine | 286.22 |  |
| HY-17477 | Guacetisal | 286.28 |  |
| HY-100949 | EC330 | 462.57 |  |
| HY-101405 | Gestrinone | 308.41 |  |
| Catalog No. | Product name | M.Wt | |
| HY-114426A | AT-1002 (TFA) | 821.91 |  |
| HY-108586 | NS3623 | 427.18 |  |
| HY-116217 | 5-Fluoro-2'-deoxycytidine | 245.21 |  |
| HY-14763A | Cariprazine (hydrochloride) | 463.87 |  |
| HY-101963 | AD80 | 473.43 |  |
| HY-100488 | Bay 59-3074 | 453.36 |  |
| HY-104032 | Ac-CoA Synthase Inhibitor1 | 410.51 |  |
| HY-112780 | UC2288 | 481.82 |  |
| HY-110177 | SP-100030 | 437.65 |  |
| HY-17580 | Fidaxomicin | 1058.04 |  |
| HY-111408 | EOAI3402143 | 503.42 |  |
| HY-112474 | Tyrphostin AG 112 | 236.23 |  |
| HY-107486 | Nosiheptide | 1222.36 |  |
| HY-112461A | NF449 (octasodium) | 1505.09 |  |
| HY-101979 | Numidargistat | 287.12 |  |
| HY-114410 | CCI-006 | 332.33 |  |
| HY-100227 | E 64c | 314.38 |  |
| HY-12821 | AEBSF (hydrochloride) | 239.69 |  |
| HY-101873 | Atorvastatin lactone | 540.62 |  |
| HY-102087 | JPM-OEt | 392.45 |  |
| HY-111341 | AZD5904 | 252.29 |  |
| HY-18081 | PF 750 | 345.44 |  |
| HY-10895 | SB-334867 | 355.78 |  |
| HY-17021 | Esomeprazole | 345.42 |  |
| HY-101418 | JNJ-47965567 | 488.64 |  |
| HY-17461A | Cortisone acetate | 402.48 |  |
| HY-10326 | GW788388 | 425.48 |  |
| HY-113973 | LPA2 antagonist 2 | 380.35 |  |
| HY-136848 | SM1-71 | 463.96 |  |
| HY-117043 | GRL0617 | 304.39 |  |
| HY-10896 | JNJ-10397049 | 484.18 |  |
| HY-100350 | CA-074 methyl ester | 397.47 |  |
| HY-110127 | NU9056 | 232.37 |  |
| HY-14545 | Amisulpride | 369.48 |  |
| HY-111342 | HDAC8-IN-1 | 345.39 |  |
| HY-14879A | Avibactam (sodium) | 287.23 |  |
| HY-114277 | Sotorasib | 560.59 |  |
| HY-103021 | LY3200882 | 435.52 |  |
| HY-17505 | Candesartan Cilexetil | 610.66 |  |
| HY-15036 | Diclofenac | 296.15 |  |
| HY-13632 | Exemestane | 296.40 |  |
| HY-105932 | Lexithromycin | 762.97 |  |
| HY-113951 | Prolyl Endopeptidase Inhibitor 1 | 296.36 |  |
| HY-14539 | Clozapine | 326.82 |  |
| HY-100593 | Spiramycin | 843.05 |  |
| HY-10454 | Delanzomib | 413.28 |  |
| HY-109078 | Vecabrutinib | 529.92 |  |
| HY-17480 | Bendazac | 282.29 |  |
| Catalog No. | Product name | M.Wt | |
| HY-17572A | Atosiban (acetate) | 1054.24 |  |
| HY-101564 | Onametostat | 483.36 |  |
| HY-10544 | Tegobuvir | 517.40 |  |
| HY-17464 | Cilostazol | 369.46 |  |
| HY-17380 | (S)-Timolol (Maleate) | 432.49 |  |
| HY-13404 | Capmatinib | 412.42 |  |
| HY-14566 | Donepezil | 379.49 |  |
| HY-15315 | Baricitinib | 371.42 |  |
| HY-N6775 | Sonolisib | 525.59 |  |
| HY-107795 | Benorilate | 313.30 |  |
| HY-104015 | NecroX-5 | 645.81 |  |
| HY-10977 | Tivozanib | 454.86 |  |
| HY-15321 | Etoricoxib | 358.84 |  |
| HY-10521 | Darapladib | 666.77 |  |
| HY-108466 | Ro 08-2750 | 270.24 |  |
| HY-15461 | Ertugliflozin | 436.88 |  |
| HY-100580 | Asaraldehyde | 196.20 |  |
| HY-108937 | NSC 15364 | 242.28 |  |
| HY-114395 | NVS-ZP7-4 | 501.62 |  |
| HY-17426 | Famciclovir | 321.33 |  |
| HY-13904 | Flumatinib | 562.59 |  |
| HY-17375 | Allylestrenol | 300.48 |  |
| HY-15440A | Fostemsavir | 583.49 |  |
| HY-109010 | Poseltinib | 470.52 |  |
| HY-14664A | Fluvastatin (sodium) | 433.45 |  |
| HY-100034 | NSC 663284 | 321.76 |  |
| HY-108003 | MM 07 | 1539.89 |  |
| HY-109061 | Lazertinib | 554.64 |  |
| HY-10533 | Eniluracil | 136.11 |  |
| HY-10572 | Efavirenz | 315.68 |  |
| HY-16060 | Apalutamide | 477.43 |  |
| HY-32718 | Pelitinib | 467.92 |  |
| HY-19915 | Contezolid | 408.33 |  |
| HY-114397 | SHP394 | 470.51 |  |
| HY-D0843 | N-Ethylmaleimide | 125.13 |  |
| HY-13553 | Anidulafungin | 1140.24 |  |
| HY-107329 | Cefathiamidine | 472.58 |  |
| HY-13571A | Beclometasone dipropionate | 521.04 |  |
| HY-A0091A | Pargyline | 159.23 |  |
| HY-121144 | Cefazedone | 548.44 |  |
| HY-50667 | Apixaban | 459.50 |  |
| HY-112608 | CHMFL-PI3KD-317 | 494.03 |  |
| HY-14171 | Bexarotene | 348.48 |  |
| HY-17600 | Acalabrutinib | 465.51 |  |
| HY-13677 | 6-Mercaptopurine | 152.18 |  |
| HY-10225 | Belinostat | 318.35 |  |
| HY-12015 | Iniparib | 292.03 |  |
| HY-10466 | Daclatasvir | 738.88 |  |
| Catalog No. | Product name | M.Wt | |
| HY-13604 | Cyproterone acetate | 402.91 |  |
| HY-103085 | T-3775440 hydrochloride | 346.85 |  |
| HY-12542A | Dantrolene (sodium hemiheptahydrate) | 399.31 |  |
| HY-13929 | Isocarboxazid | 231.25 |  |
| HY-17506 | Azithromycin | 748.98 |  |
| HY-12872 | Nazartinib | 495.02 |  |
| HY-14144 | Aclidinium (Bromide) | 564.55 |  |
| HY-13636 | Fulvestrant | 606.77 |  |
| HY-14737 | Ceftaroline fosamil | 744.74 |  |
| HY-100675 | JTE-013 | 408.29 |  |
| HY-17431 | Fosamprenavir (Calcium Salt) | 623.67 |  |
| HY-10234 | Saracatinib | 542.03 |  |
| HY-10237 | Boceprevir | 519.68 |  |
| HY-15306 | Eltrombopag | 442.47 |  |
| HY-14398 | Celecoxib | 381.37 |  |
| HY-17001 | Flupirtine (Maleate) | 420.39 |  |
| HY-107824 | D-Melibiose | 342.30 |  |
| HY-14434 | Asunaprevir | 748.29 |  |
| HY-14546 | Aripiprazole | 448.39 |  |
| HY-A0004 | Decitabine | 228.21 |  |
| HY-17592 | Bithionol | 356.05 |  |
| HY-112274 | M443 | 589.61 |  |
| HY-101509 | HSL-IN-1 | 436.58 |  |
| HY-14946 | Amifampridine | 109.13 |  |
| HY-122720 | SEC inhibitor KL-1 | 345.78 |  |
| HY-107496 | Imidocarb (dipropionate) | 496.56 |  |
| HY-17573 | Carbetocin | 988.16 |  |
| HY-12785 | Albendazole sulfoxide | 281.33 |  |
| HY-A0061 | Trifluridine | 296.20 |  |
| HY-100753 | STAT3-IN-1 | 475.53 |  |
| HY-101478 | Fenobam | 266.68 |  |
| HY-107459 | (E/Z)-AG490 | 294.30 |  |
| HY-115584 | Lufenuron | 511.15 |  |
| HY-17042A | Cetirizine (dihydrochloride) | 461.81 |  |
| HY-13463 | Avatrombopag | 649.65 |  |
| HY-17469 | Gimeracil | 145.54 |  |
| HY-101712 | TRC051384 | 465.54 |  |
| HY-112906 | C-176 | 358.09 |  |
| HY-112596 | H3B-6545 | 567.58 |  |
| HY-108330 | AG126 | 215.17 |  |
| HY-12823 | BLU9931 | 509.38 |  |
| HY-19834 | Fenebrutinib | 664.80 |  |
| HY-16952A | Bepridil (hydrochloride hydrate) | 421.02 |  |
| HY-122537A | Arotinolol | 371.54 |  |
| HY-138565 | K-975 | 287.74 |  |
| HY-114454 | INH14 | 240.30 |  |
| HY-13734 | PX-12 | 188.31 |  |
| HY-11021 | Elinogrel | 523.95 |  |
| Catalog No. | Product name | M.Wt | |
| HY-101736 | AMG9810 | 337.41 |  |
| HY-128922 | Dexamethasone palmitate | 630.87 |  |
| HY-16767 | Doravirine | 425.75 |  |
| HY-109189 | Rezivertinib | 486.57 |  |
| HY-101426 | RG14620 | 275.13 |  |
| HY-12085 | Apremilast | 460.50 |  |
| HY-13551 | Amsacrine | 393.46 |  |
| HY-100877 | GCN2-IN-1 | 402.41 |  |
| HY-112570 | CC-90003 | 458.44 |  |
| HY-15461A | Ertugliflozin L-pyroglutamic acid | 566.00 |  |
| HY-10261A | Afatinib (dimaleate) | 718.08 |  |
| HY-135815 | Mobocertinib | 585.70 |  |
| HY-107411 | Bromoenol lactone | 317.18 |  |
| HY-101908 | BMS CCR2 22 | 593.66 |  |
| HY-12214A | NVP-2 | 513.07 |  |
| HY-100631 | FGFR4-IN-1 | 493.52 |  |
| HY-111540A | (Rac)-LY-3381916 | 396.45 |  |
| HY-18342 | Diflunisal | 250.20 |  |
| HY-12533 | Disopyramide | 339.47 |  |
| HY-109086 | Edicotinib | 461.60 |  |
| HY-13943 | CNX-774 | 499.50 |  |
| HY-13896 | PD168393 | 369.22 |  |
| HY-107371 | 6-Bromo-2-hydroxy-3-methoxybenzaldehyde | 231.04 |  |
| HY-103310 | MRS1845 | 398.41 |  |
| HY-107814 | Nicarbazin | 426.38 |  |
| HY-12720A | Apraclonidine (hydrochloride) | 281.57 |  |
| HY-10978 | Crisaborole | 251.05 |  |
| HY-14200 | (S)-Rasagiline | 171.24 |  |
| HY-13575 | Blonanserin | 367.50 |  |
| HY-100578 | AGK2 | 434.27 |  |
| HY-116100A | HA155 | 463.29 |  |
| HY-W012875 | 3-Nitropropanoic acid | 119.08 |  |
| HY-112166 | Rilzabrutinib | 665.76 |  |
| HY-13605A | Cytarabine (hydrochloride) | 279.68 |  |
| HY-14654 | Aspirin | 180.16 |  |
| HY-18012 | Spebrutinib | 423.44 |  |
| HY-112157 | PF-06751979 | 455.50 |  |
| HY-N0191 | Andrographolide | 350.45 |  |
| HY-B1199 | Nialamide | 298.34 |  |
| HY-15789 | Elbasvir | 882.02 |  |
| HY-17040 | Darunavir | 547.66 |  |
| HY-B0431A | Phenoxybenzamine (hydrochloride) | 340.29 |  |
| HY-17396 | Butenafine (Hydrochloride) | 353.93 |  |
| HY-17586 | Dalbavancin (hydrochloride) | 1853.15 |  |
| HY-123963 | C-178 | 322.27 |  |
| HY-112076 | Atropine methyl (bromide) | 384.31 |  |
| HY-13207 | ONX-0914 | 580.67 |  |
| HY-12213 | CDDO-EA | 518.73 |  |
| Catalog No. | Product name | M.Wt | |
| HY-80013 | THZ1 | 566.05 |  |
| HY-52101A | FMK | 342.37 |  |
| HY-128867 | bio-THZ1 | 1053.67 |  |
| HY-121203 | Citalopram | 324.39 |  |
| HY-112398 | GSK1379725A | 450.47 |  |
| HY-15714 | NMS-859 | 349.79 |  |
| HY-13016 | Cabozantinib | 501.51 |  |
| HY-15780 | Brexpiprazole | 433.57 |  |
| HY-10274 | Dabigatran etexilate | 627.73 |  |
| HY-111457A | BAY-678 | 400.35 |  |
| HY-116035 | Nimbolide | 466.52 |  |
| HY-100399A | Nevanimibe hydrochloride | 458.08 |  |
| HY-10325 | CL-387785 | 381.23 |  |
| HY-101481 | Flurbiprofen axetil | 330.35 |  |
| HY-15781A | (R)-Morinidazole | 270.29 |  |
| HY-B0886A | Iproniazid | 179.22 |  |
| HY-17427 | Emtricitabine | 247.25 |  |
| HY-12305 | Q-VD-OPh | 513.49 |  |
| HY-108588 | NS5806 | 574.07 |  |
| HY-109015 | Tucidinostat | 390.41 |  |
| HY-17465 | Glycopyrrolate | 398.33 |  |
| HY-117604 | THPP-1 | 464.90 |  |
| HY-17498 | Atenolol | 266.34 |  |
| HY-112823 | Almonertinib | 525.64 |  |
| HY-107319 | Almitrine mesylate | 669.76 |  |
| HY-14872 | Tideglusib | 334.39 |  |
| HY-18603 | FIIN-3 | 691.61 |  |
| HY-100546A | GSK-LSD1 (dihydrochloride) | 289.24 |  |
| HY-112693 | H-151 | 279.34 |  |
| HY-100529 | PD150606 | 306.12 |  |
| HY-15605 | Encorafenib | 540.01 |  |
| HY-117102 | ANI-7 | 263.12 |  |
| HY-10261 | Afatinib | 485.94 |  |
| HY-110192 | ML 297 | 328.32 |  |
| HY-107589 | BIO5192 | 817.78 |  |
| HY-15249 | JZL 184 | 520.49 |  |
| HY-14914 | Azilsartan | 456.45 |  |
| HY-17385 | Atomoxetine (hydrochloride) | 291.82 |  |
| HY-13613 | Dutasteride | 528.53 |  |
| HY-17359 | Deferasirox | 373.36 |  |
| HY-17356 | Fenofibrate | 360.83 |  |
| HY-112611 | H3B-5942 | 494.63 |  |
| HY-100003 | ML-210 | 475.32 |  |
| HY-10493 | Cobicistat | 776.02 |  |
| HY-107867 | (±)-Clopidogrel (bisulfate) | 419.90 |  |
| HY-15771 | Tirabrutinib | 454.48 |  |
| HY-13625 | Ertapenem sodium | 497.50 |  |
| HY-W018161 | Hexadecanedioic acid | 286.41 |  |
| Catalog No. | Product name | M.Wt | |
| HY-32721 | Neratinib | 557.04 |  |
| HY-108465 | Pyr3 | 456.63 |  |
| HY-111540 | LY-3381916 | 396.45 |  |
| HY-136605 | DGY-06-116 | 597.11 |  |
| HY-17367 | Atazanavir | 704.86 |  |
| HY-10498 | Lexibulin | 434.53 |  |
| HY-16973 | Fluralaner | 556.29 |  |
| HY-10367 | Canertinib | 485.94 |  |
| HY-13600 | Clobetasol propionate | 466.97 |  |
| HY-14736 | Azilsartan medoxomil | 568.53 |  |
| HY-116506 | Bigelovin | 304.34 |  |
| HY-107412 | Proteasome inhibitor IX | 377.01 |  |
| HY-103097 | ST271 | 272.34 |  |
| HY-111430 | 1-Ethynylnaphthalene | 152.19 |  |
| HY-13038A | Fostamatinib | 580.46 |  |
| HY-17468 | Bumetanide | 364.42 |  |
| HY-10331 | Regorafenib | 482.82 |  |
| HY-101192 | GSK682753A | 479.78 |  |
| HY-10230 | Midostaurin | 570.64 |  |
| HY-115496 | 7-BIA | 294.30 |  |
| HY-111457 | BAY-677 | 400.35 |  |
| HY-107385 | Epristeride | 399.57 |  |
| HY-14605A | Rasagiline | 171.24 |  |
| HY-14740 | Elvitegravir | 447.88 |  |
| HY-10227 | Bortezomib | 384.24 |  |
| HY-10210 | Retaspimycin (hydrochloride) | 624.17 |  |
| HY-14645 | (–)-DHMEQ | 261.23 |  |
| HY-101568 | Roblitinib | 506.56 |  |
| HY-12176 | Aliskiren | 551.76 |  |
| HY-17376 | Ezetimibe | 409.43 |  |
| HY-10320 | Doramapimod | 527.66 |  |
| HY-14289 | Cimetidine | 252.34 |  |
| HY-17404 | Cilnidipine | 492.52 |  |
| HY-18252 | Avanafil | 483.95 |  |
| HY-100584 | Davercin | 759.92 |  |
| HY-50895 | Gefitinib | 446.90 |  |
| HY-10224 | Panobinostat | 349.43 |  |
| HY-14598 | Diethylstilbestrol | 268.35 |  |
| HY-17501A | Bambuterol hydrochloride | 403.90 |  |
| HY-100229 | Aloxistatin | 342.43 |  |
| HY-100574A | Cl-amidine (hydrochloride) | 347.24 |  |
| HY-B0334 | Sulbactam | 233.24 |  |
| HY-100754 | Ritlecitinib | 285.34 |  |
| HY-100818 | Futibatinib | 418.45 |  |
| HY-13619A | Efaproxiral (sodium) | 363.38 |  |
| HY-12005 | Fingolimod (hydrochloride) | 343.93 |  |
| HY-112823A | Almonertinib (mesylate) | 621.75 |  |
| HY-102046 | FM-381 | 428.49 |  |
| Catalog No. | Product name | M.Wt | |
| HY-101976 | JAK3-IN-6 | 350.37 |  |
| HY-16985 | Darolutamide | 398.85 |  |
| HY-17038 | Agomelatine | 243.30 |  |
| HY-14149 | Cisapride | 465.95 |  |
| HY-10285 | Saxagliptin | 315.41 |  |
| HY-18716 | Dextrorotation nimorazole phosphate ester | 350.26 | |
| HY-10052 | Aprepitant | 534.43 |  |
| HY-101280 | LB-60-OF61 | 494.59 |  |
| HY-136351 | THZ-P1-2 | 531.61 |  |
| HY-13571 | Betamethasone dipropionate | 504.59 |  |
| HY-19985 | PF-06459988 | 431.88 |  |
| HY-14603 | Clioquinol | 305.50 |  |
| HY-10586 | 5-Azacytidine | 244.20 |  |
| HY-112161 | Branebrutinib | 370.42 |  |
| HY-15206 | Glibenclamide | 494.00 |  |
| HY-122872 | MKK7-COV-9 | 320.35 |  |
| HY-10257 | BAY 11-7085 | 249.33 |  |
| HY-10341 | Fasudil (Hydrochloride) | 327.83 |  |
| HY-19912 | Fruquintinib | 393.39 |  |
| HY-18300 | Filgotinib | 425.50 |  |
| HY-15656 | Ceritinib | 558.14 |  |
| HY-14249 | Bicalutamide | 430.37 |  |
| HY-103095 | Enmetazobactam | 314.32 |  |
| HY-101842 | ND-646 | 568.64 |  |
| HY-U00447 | PK11000 | 236.63 |  |
| HY-10446 | Pralatrexate | 477.47 |  |
| HY-17423 | Abacavir | 286.33 |  |
| HY-10574 | Rilpivirine | 366.42 |  |
| HY-B1235 | Acetohydroxamic acid | 75.07 |  |
| HY-17361 | Etofenamate | 369.34 |  |
| HY-100510 | RAF709 | 542.55 |  |
| HY-10582 | Flurbiprofen | 244.26 |  |
| HY-18602 | FIIN-2 | 634.73 |  |
| HY-10882 | Clotrimazole | 344.84 |  |
| HY-A0006 | Pentostatin | 268.27 |  |
| HY-108402A | Cefodizime (sodium) | 628.63 |  |
| HY-19816A | Avitinib (maleate) | 603.60 |  |
| HY-106353 | Smilagenin | 416.64 |  |
| HY-B0744 | Eflornithine | 182.17 |  |
| HY-108402 | Cefodizime | 584.67 |  |
| HY-124704 | Chst15-IN-1 | 442.09 |  |
| HY-13585 | Carmustine | 214.05 |  |
| HY-12972 | Mavelertinib | 415.42 |  |
| HY-15592 | Cabotegravir | 405.35 |  |
| HY-13570 | Betamethasone | 392.46 |  |
| HY-15202 | Binimetinib | 441.23 |  |
| HY-110243 | CAIX Inhibitor S4 | 335.38 |  |
| HY-14274 | Anastrozole | 293.37 |  |
| Catalog No. | Product name | M.Wt | |
| HY-117163 | FzM1.8 | 322.31 |  |
| HY-15234 | Fluticasone furoate | 538.58 |  |
| HY-130149 | Adagrasib | 604.12 |  |
| HY-17476 | Carbasalate calcium | 259.24 |  |
| HY-50878 | Crizotinib | 450.34 |  |
| HY-108365 | Gamithromycin | 777.04 |  |
| HY-16677A | Mofegiline (hydrochloride) | 233.69 |  |
| HY-100223 | Calpeptin | 362.46 |  |
| HY-112847A | Sulfosuccinimidyl oleate (sodium) | 481.58 |  |
| HY-10065 | Axitinib | 386.47 |  |
| HY-10932 | Aniracetam | 219.24 |  |
| HY-111783 | AZD-7648 | 380.40 |  |
| HY-103714A | Ensartinib (dihydrochloride) | 634.36 |  |
| HY-114226 | Olutasidenib | 354.79 |  |
| HY-107212 | Selamectin | 769.96 |  |
| HY-17495A | Carteolol hydrochloride | 328.8343 |  |
| HY-114436 | MRTX-1257 | 565.71 |  |
| HY-10004 | Faropenem daloxate | 397.40 |  |
| HY-131704 | FGFR4-IN-5 | 520.37 |  |
| HY-101813 | Laflunimus | 310.27 |  |
| HY-101560 | Linrodostat | 410.91 |  |
| HY-101042 | AG-494 | 280.28 |  |
| HY-100962 | (E)-AG 99 | 204.19 |  |
| HY-102033 | Oxamflatin | 342.37 |  |
| HY-100222 | CZ415 | 459.56 |  |
| HY-15577 | GSK3787 | 392.78 |  |
| HY-108411 | Emedastine | 302.41 |  |
| HY-10346 | AV-412 | 851.41 |  |
| HY-108974 | Drotaverine (hydrochloride) | 433.97 |  |
| HY-101267 | CHMFL-BMX-078 | 625.67 |  |
| HY-13036A | IBT6A | 386.45 |  |
| HY-19750 | TBA-7371 | 355.39 |  |
| HY-14881 | Bedaquiline | 555.50 |  |
| HY-10181 | Dasatinib | 488.01 |  |
| HY-111823 | VH032 thiol | 490.64 |  |
| HY-111959 | 2-Selenouracil | 175.05 |  |
| HY-108556A | RWJ-56110 (dihydrochloride) | 863.65 |  |
| HY-13897 | CNX-2006 | 545.53 |  |
| HY-114409 | GB1107 | 522.32 |  |
| HY-101766 | Btk inhibitor 2 | 431.49 |  |
| HY-101298 | Paprotrain | 245.28 |  |
| HY-10253 | AG1024 | 305.17 |  |
| HY-17605 | Bictegravir | 449.38 |  |
| HY-101708 | N-(2-Chloro-6-methylphenyl)-N′-4-pyridinylurea | 261.71 | |
| HY-101429 | RG13022 | 266.29 |  |
| HY-105129 | Pimonidazole (hydrochloride) | 290.75 |  |
| HY-101503 | HTS01037 | 337.37 |  |
| HY-123847 | KPT-6566 | 443.54 |  |
| Catalog No. | Product name | M.Wt | |
| HY-11080 | PKI-179 | 488.54 |  |
| HY-115282A | JNJ-63576253 | 538.97 |  |
| HY-10398 | CTS-1027 | 425.8832 |  |
| HY-111365 | TES-1025 | 383.44 |  |
| HY-19730 | Olmutinib | 486.59 |  |
| HY-100528A | Dianemycin | 867.11 |  |
| HY-19564 | JX06 | 324.51 |  |
| HY-117113 | JI051 | 364.44 |  |
| HY-110237 | BX430 | 413.11 |  |
| HY-116152 | Cipepofol | 204.31 |  |
| HY-103350 | CA-074 | 383.44 |  |
| HY-80002 | BMX-IN-1 | 524.59 |  |
| HY-101457 | JZP-430 | 354.47 |  |
| HY-101131 | Dansyl glutathione | 540.61 |  |
| HY-117570 | KSC-34 | 429.95 |  |
| HY-10121 | Asenapine | 285.77 |  |
| HY-112720 | AGL-2263 | 322.27 |  |
| HY-112292 | GW806742X | 573.55 |  |
| HY-100705 | DMNB | 211.17 |  |
| HY-104051 | Monacolin J | 320.42 |  |
| HY-100499 | Tyrphostin AG 528 | 306.32 |  |
| HY-103443 | HKI-357 | 574.05 |  |
| HY-122054A | BPK-29 (hydrochloride) | 506.46 |  |
| HY-10453 | Ixazomib | 361.03 |  |
| HY-18632 | GSK2879552 | 364.48 |  |
| HY-110404 | Capzimin | 628.81 |  |
| HY-108435 | GNE-049 | 510.58 |  |
| HY-103286 | PD176252 | 584.67 |  |
| HY-B0496 | PMSF | 174.19 |  |
| HY-128892 | EN6 | 368.34 |  |
| HY-100492 | Fisogatinib | 503.38 |  |
| HY-14879 | Avibactam (free acid) | 265.24 |  |
| HY-101474 | (±)-Zanubrutinib | 471.55 |  |
| HY-112423 | CE-245677 | 513.38 |  |
| HY-13509 | CCG-50014 | 316.35 |  |
| HY-103129 | SB-200646A | 302.76 |  |
| HY-106574A | Ceftobiprole medocaril (sodium) | 712.64 |  |
| HY-10408 | Ki20227 | 480.54 |  |
| HY-17646 | Verdiperstat | 253.32 |  |
| HY-127104 | FMF-04-159-2 | 683.01 |  |
| HY-101786 | KZR-504 | 413.42 |  |
| HY-110159 | (E/Z)-Teriflunomide | 270.21 |  |
| HY-10218 | Everolimus | 958.22 |  |
| HY-10396 | Emricasan | 569.50 |  |
| HY-101868 | INF39 | 224.68 |  |
| HY-50202 | Etomoxir | 326.82 |  |
| HY-112671 | CDDO-dhTFEA | 574.72 |  |
| HY-114395A | (R)-NVS-ZP7-4 | 501.62 |  |
| Catalog No. | Product name | M.Wt | |
| HY-111532 | (3R,4R)-A2-32-01 | 301.42 |  |
| HY-125006 | TRi-1 | 328.73 |  |
| HY-126247 | BI-2852 | 516.59 |  |
| HY-115051 | 2-Hydroxymethyltetrahydropyran | 116.16 |  |
| HY-100573 | Necrosulfonamide | 461.47 |  |
| HY-10238 | Danoprevir | 731.83 |  |
| HY-15298A | Grazoprevir potassium salt | 804.99 |  |
| HY-14885 | Eliglustat | 404.54 |  |
| HY-107782 | Picrotin | 310.30 |  |
| HY-10633 | SB-705498 | 429.23 |  |
| HY-18930 | NU6300 | 413.49 |  |
| HY-101768 | PRN1371 | 561.46 |  |
| HY-102064 | SR 57227A | 248.15 |  |
| HY-112937 | GNE-6640 | 330.38 |  |
| HY-101844 | ML-18 | 569.65 |  |
| HY-113110A | Cysteinylglycine (TFA) | 292.23 |  |
| HY-12903 | Macozinone | 456.48 |  |
| HY-108017 | Ferric maltol | 431.15 |  |
| HY-100244 | NS1652 | 324.25 |  |
| HY-135221 | Cefcapene pivoxil (hydrochloride) | 604.10 |  |
| HY-15298B | Grazoprevir hydrate | 784.92 |  |
| HY-133740 | MS117 | 298.38 |  |
| HY-116835 | BI-6901 | 453.56 |  |
| HY-112215 | ARQ 531 | 478.93 |  |
| HY-10499 | PH-064 | 795.11 |  |
| HY-101567 | BMS-986158 | 495.62 |  |
| HY-10219 | Rapamycin | 914.17 |  |
| HY-19729 | Naquotinib | 562.71 |  |
| HY-100965 | Diphenyleneiodonium chloride | 314.55 |  |
| HY-113402A | Gamma-glutamylcysteine (TFA) | 364.30 |  |
| HY-14814 | Delafloxacin | 440.76 |  |
| HY-114348 | N,N'-Diacetyl-L-cystine | 324.37 |  |
| HY-101396 | ICA-069673 | 269.63 |  |
| HY-113466 | 4-Hydroxynonenal | 156.22 |  |
| HY-100495 | FT011 | 351.35 |  |
| HY-107455 | A-485 | 536.48 |  |
| HY-10389 | Alvespimycin | 616.75 |  |
| HY-110063 | 4-IPP | 282.08 |  |
| HY-111664A | (S)​-​(-​)​-​Citronellal | 154.25 |  |
| HY-111790 | M3258 | 329.16 |  |
| HY-14380 | PF-3845 | 456.46 |  |
| HY-100528 | Nanchangmycin | 889.10 |  |
| HY-109187A | Posenacaftor (sodium) | 467.49 |  |
| HY-100898 | OGT 2115 | 495.30 |  |
| HY-117203A | CDK12-IN-E9 | 434.53 |  |
| HY-114453 | SHP389 | 484.98 |  |
| HY-111664 | (R)-(+)-Citronellal | 154.25 |  |
| HY-10559 | Nelotanserin | 437.24 |  |
| Catalog No. | Product name | M.Wt | |
| HY-104066 | Theliatinib | 442.51 |  |
| HY-112619 | TES-991 | 393.45 |  |
| HY-100973A | Adenosine 5′-diphosphoribose (sodium) | 581.30 |  |
| HY-100739 | RA190 | 596.76 |  |
| HY-117491 | BRD4 Inhibitor-10 | 429.51 |  |
| HY-10981 | Lenvatinib | 426.85 |  |
| HY-111553 | TAS0728 | 504.58 |  |
| HY-114357A | DS-1205b (free base) | 735.80 |  |
| HY-100433 | PACMA 31 | 430.47 |  |
| HY-16596 | CNX-1351 | 573.71 |  |
| HY-15813 | FIIN-1 | 656.60 |  |
| HY-15298 | Grazoprevir | 766.90 |  |
| HY-13209 | Ambrisentan | 378.42 |  |
| HY-17430 | Amprenavir | 505.63 |  |
| HY-13011 | Alectinib | 482.62 |  |
| HY-14268 | Febuxostat | 316.37 |  |
| HY-13580 | Budesonide | 430.53 |  |
| HY-14188 | Amiodarone (hydrochloride) | 681.77 |  |
| HY-101016 | 17-ODYA | 280.45 |  |
| HY-17634 | Glecaprevir | 838.87 |  |
| HY-10451 | Canagliflozin | 444.52 |  |
| HY-12719 | Dexmedetomidine | 200.28 |  |
| HY-104040 | MKC8866 | 361.35 |  |
| HY-103389 | 1-Aminobenzotriazole | 134.14 |  |
| HY-14197A | Clorgyline hydrochloride | 308.63 |  |
| HY-101980 | Imaradenant | 315.73 |  |
| HY-13637 | Ganciclovir | 255.23 |  |
| HY-10158 | Bosutinib | 530.45 |  |
| HY-B1496 | Tranylcypromine (hemisulfate) | 182.23 |  |
| HY-17508 | Clarithromycin | 747.95336 |  |
| HY-13238 | Dolutegravir | 419.38 |  |
| HY-15772 | Osimertinib | 499.61 |  |
| HY-10264 | Edoxaban | 548.06 |  |
| HY-111603 | Calcium dobesilate | 418.41 |  |
| HY-13064 | Cobimetinib | 531.31 |  |
| HY-13599 | Cladribine | 285.69 |  |
| HY-10223 | CUDC-101 | 434.49 |  |
| HY-B0218 | Orlistat | 495.73 |  |
| HY-50896 | Erlotinib | 393.44 |  |
| HY-114657A | Benproperine (phosphate) | 407.44 |  |
| HY-14280 | Entacapone | 305.29 |  |
| HY-17026 | Gemcitabine | 263.20 |  |
| HY-13272 | Dacomitinib | 469.94 |  |
| HY-107855 | DL-Mevalonolactone | 130.14 |  |
| HY-75054 | Abiraterone acetate | 391.55 |  |
| HY-105685 | SRX246 | 703.87 |  |
| HY-10201 | Sorafenib | 464.83 |  |
| HY-10211 | Tanespimycin | 585.69 |  |
| Catalog No. | Product name | M.Wt | |
| HY-15282 | E-64 | 357.41 |  |
| HY-14660 | Dabrafenib | 519.56 |  |
| HY-15409 | Empagliflozin | 450.91 |  |
| HY-13998 | Dasabuvir | 493.57 |  |
| HY-16297 | Abemaciclib (methanesulfonate) | 602.70 |  |
| HY-13624A | Epirubicin (hydrochloride) | 579.98 |  |
| HY-112306 | Ripretinib | 510.36 |  |
| HY-114778 | Fluzoparib | 472.40 |  |
| HY-15816 | Ulixertinib | 433.33 |  |
| HY-15283 | Clopidogrel | 321.82 |  |
| HY-17379 | Atorvastatin (hemicalcium salt) | 577.67 |  |
| HY-109025 | Baloxavir marboxil | 571.55 |  |
| HY-10997 | Ibrutinib | 440.50 |  |
| HY-17422 | Acyclovir | 225.20 |  |
| HY-17461 | Cortisone | 360.44 |  |
| HY-14648A | Dexamethasone acetate | 434.50 |  |
| HY-17405 | Alarelin (Acetate) | 1287.42 |  |
| HY-13229 | BOC-D-FMK | 263.26 |  |
| HY-14648 | Dexamethasone | 392.46 |  |
| HY-13635 | Finasteride | 372.54 |  |
| HY-10450 | Dapagliflozin | 408.87 |  |
| HY-15399 | Vigabatrin | 129.16 |  |
| HY-12782T | Iadademstat (dihydrochloride) | 303.27 |  |
| HY-106376A | L-Buthionine-(S,R)-sulfoximine | 222.31 |  |
| HY-100381 | Nigericin (sodium salt) | 746.94 |  |
